# The effects of a newly developed beverage powder supplement on anthropometric measures, body composition, metabolic syndrome components, and appetite in obese or overweight adults: a protocol for a randomized clinical trial study

**DOI:** 10.1186/s13063-025-09180-3

**Published:** 2025-10-29

**Authors:** Parastoo Asghari, Asma Afshari, Tahereh Sadeghi, Ali Jafarzadeh Esfehani, Saeedeh Talebi, Mohsen Nematy

**Affiliations:** 1 Department of Nutrition, Faculty of Medicine, Mashhad University of Medical Sciences, Mashhad, Iran; 2 School of Nursing and Midwifery, Nursing and Midwifery Care Research Center, Akbar Hospital, Mashhad University of Medical Sciences, Mashhad, Iran; 3 Metabolic Syndrome Research Center, Mashhad University of Medical Sciences, The University Campus (Paradise Daneshgah), Azadi Square, Mashhad, 91779-48564 Iran

**Keywords:** *Akkermansia muciniphila*, Green tea extract, Konjac glucomannan, Next generation probiotics, Obesity

## Abstract

**Background:**

Overweight and obesity result from excessive fat accumulation and pose significant global health challenges linked to various diseases. Recent research suggests that gut microbiota, particularly dysbiosis, is vital in obesity development. Next-generation probiotics, especially *Akkermansia muciniphila*, alongside compounds found in green tea extract and konjac glucomannan, offer promise in obesity management by enhancing gut microbiota balance and metabolic function. This research investigates a unique supplement that integrates these elements, proposing that it will outperform lifestyle changes alone in improving anthropometric measures, body composition, metabolic metrics, blood pressure, and appetite among overweight or obese adults.

**Methods:**

This study will include 72 overweight or obese patients, who will be randomly assigned to either the intervention or control group within a randomized, triple-blind controlled clinical trial. The intervention group will consume 2 g of beverage sachets containing pasteurized *Akkermansia muciniphila*, green tea extract, and konjac glucomannan three times daily. In comparison, the control group will take 2 g of microcrystalline cellulose three times daily for 8 weeks. Evaluations of anthropometry (body mass index, waist and hip circumference), body composition (fat mass, fat-free mass, total body water, etc.), appetite using a visual analogue scale, blood pressure, and biochemical parameters including fasting blood glucose (FBS), total cholesterol (TC), triglycerides (TG), low- and high-density lipoprotein (LDL and HDL) will be conducted.

**Discussion:**

The results of this study will provide evidence for the synergistic effects of *Akkermansia muciniphila* combined with prebiotics in the management of obesity and its associated metabolic states.

**Trial registration:**

Iranian Registry of Clinical Trials (IRCT20250222064807N2) (2025/03/26).

**Supplementary Information:**

The online version contains supplementary material available at 10.1186/s13063-025-09180-3.

## Introduction

### Background and rationale {6a}

Overweight and obesity are acknowledged as global health issues [1]. Obesity is defined as abnormal or excessive fat accumulation that may be harmful to health [2]. The World Health Organization (WHO) defines overweight individuals as those with a BMI between 25 and 29.9 kg/m^2^, whereas obesity is indicated by a BMI of 30 kg/m^2^ or more. Obesity is further divided into three classes: class I (BMI = 30–34.9 kg/m^2^), class II (35–39.9 kg/m^2^), and class III (≥ 40 kg/m^2^) [3]. Being obese heightens the risk of numerous diseases and conditions, which are associated with a higher risk of mortality. These include yype 2 diabetes mellitus (T2DM), cardiovascular diseases (CVD), metabolic syndrome (MetS), chronic kidney disease (CKD), elevated lipid levels, high blood pressure, nonalcoholic fatty liver disease (NAFLD), certain cancer types, obstructive sleep apnea, osteoarthritis, and depression [4]. Obesity rates are increasing globally, and managing this issue is difficult due to its complex etiology, which involves various contributing factors such as poor diets, lack of physical activity, genetic predispositions, and social and environmental influences [5].

Increasing evidence indicates that gut microbiota, an important environmental factor, has a considerable influence on the onset and advancement of obesity [6]. Dysbiosis—an imbalance in the microbiome—leads to increased appetite and the development of metabolic disorders, both of which contribute to obesity and its associated issues [7]. Probiotics are live microorganisms that provide health benefits when consumed in adequate amounts [8]. Probiotics are well-known for their ability to enhance gut microbiota and combat dysbiosis [9]. Probiotics influence obesity by effectively regulating the gut’s microbial balance, reducing insulin resistance, and improving feelings of fullness [7]. Although conventional probiotics tend to have limited beneficial effects, next-generation probiotics (NGP) are now recognized as innovative preventive and therapeutic options [10].

*Akkermansia muciniphila*, a bacterium that inhabits the mucus layer of the gastrointestinal tract, has emerged as a promising next-generation probiotic for addressing obesity-related conditions [11]. *A. muciniphila* affects obesity by regulating metabolism and energy balance while also improving insulin sensitivity and glucose control [12]. *A. muciniphila* has shown an inverse relationship with the development of inflammation, disrupted adipose tissue metabolism, and metabolic disorders in animal models of obesity [13]. Depommier et al. reported that pasteurized A. *muciniphila* improved insulin sensitivity and reduced serum insulin, total cholesterol, liver function, and inflammatory biomarkers in overweight or obese, insulin-resistant volunteers compared to placebo. It also led to a slight decrease in body weight, fat mass, and hip circumference from baseline, without significantly impacting the gut microbiome composition [14].

Green tea extract is rich in various polyphenolic compounds with antioxidant benefits. However, the key active ingredients are flavanol monomers called catechins, particularly epigallocatechin-3-gallate and epicatechin-3-gallate, which are the most potent antioxidant compounds [15]. Numerous laboratory studies have indicated that the polyphenols found in green tea might affect gut microbiota, hinting at possible anti-obesity effects through the modulation of gut bacteria [16].

EGCG has been demonstrated to modify gut microbiota by promoting the growth of A. *muciniphila*, which helps improve gut dysbiosis and enhances barrier integrity. A recent laboratory study revealed that when EGCG is co-metabolized with mucin and glucose, it boosts the growth of A. *muciniphila*, further supporting the proposed mechanisms of polyphenolic prebiotic effects. This effective co-metabolism implies that incorporating EGCG with glucose-based dietary fibers could significantly increase the levels of this bacterium within the intestinal microbiota [17]. Current research indicates that GTE might aid in weight control, blood glucose regulation, and lowering total cholesterol (TC), LDL cholesterol (LDL-C), and blood pressure (BP) in overweight or obese individuals [18].

Konjac glucomannan (KGM) is a hydrocolloidal polysaccharide fiber derived from the tubers of Amorphophallus konjac. KGM offers potential health benefits due to its diverse biological activities, which include anti-diabetic, anti-obesity, laxative, prebiotic, and anti-inflammatory effects [19]. KGM can help combat type 2 diabetes, reduce obesity, and lower blood pressure and lipid levels. Its hydration capacity and enhanced viscosity promote feelings of fullness, slow down glycemic responses, and limit lipid absorption. KGM helps prevent metabolic syndrome by regulating gut microbiota [20].

### Objectives {7}

This study explores methods to prevent and manage obesity and its complications by examining a novel beverage powder supplement containing pasteurized *Akkermansia muciniphila*, green tea extract, and glucomannan konjac. Although the effects of each component have been previously investigated, the impact of combining these components has not been explored. The primary focus is on the supplement’s effects on body composition and anthropometric measurements, as well as metabolic parameters (including fasting blood glucose and serum lipid profile), blood pressure, and appetite, as secondary outcomes in overweight or obese adults. We hypothesize that this supplement, when combined with lifestyle interventions such as dietary therapy and physical activity, is more effective than lifestyle interventions alone in improving these outcome variables.

### Trial design {8}

Our study will be a randomized, triple-blind, parallel-group, superiority trial with a 1:1 allocation ratio. Overweight and obese adults will be randomly assigned to either a new beverage powder supplement or a placebo for 8 weeks, along with standard lifestyle guidance. This design enables a thorough comparison of the supplement’s effectiveness against the placebo, while accounting for potential confounding variables through blinding and randomization.

## Methods: participants, interventions and outcomes

### Study setting {9}

This randomized, triple-blind, placebo-controlled clinical trial with a parallel-group design will take place over four months, starting in June 2025, in a nutrition clinic located in Mashhad, Iran.

### Eligibility criteria {10}

#### Inclusion criteria

Adults aged 18 to 45 who are overweight or obese, with a body mass index (BMI) ranging from 27 to 34.9 kg/m^2^, and waist circumference greater than 102 cm for men and greater than 88 cm for women, who maintain a stable weight (no more than 5% weight change in the last 3 months), and provide written consent, will be recruited from a nutrition clinic in Mashhad, Iran.

### Exclusion criteria

We will exclude individuals who are pregnant, lactating, or postmenopausal; professional athletes or individuals undergoing changes in the intensity and frequency of their physical activities throughout the last month; individuals with a confirmed clinical history of eating disorders, gastrointestinal diseases, cardiovascular, hepatic, renal, pulmonary, or endocrine diseases (such as thyroid conditions or diabetes), cancer, or any severe acute or chronic disease; current smokers, drug or alcohol addicts; those with known allergies or sensitivities to supplement or placebo components; individuals with a history of bariatric surgery; those who skip at least one meal; individuals on special diets, such as vegetarian diets; those with a regular intake of more than 300 mg of caffeine or more than 30 g of fiber per day; those adhering to a calorie-restricted diet for three months prior to the start of the study; individuals who routinely consume pre-, pro-, and synbiotic products; those who take antibiotics for one month before the study begins; or individuals using medications, including cholesterol and lipid-lowering drugs (e.g., statins), blood sugar-lowering drugs (e.g., insulin, metformin, liraglutide), antihypertensive medications, drugs affecting GI function, weight, body composition, or appetite (e.g., antipsychotics, corticosteroids, anti-obesity drugs, oral contraceptives, antidepressants), weight loss supplements (including those affecting appetite, metabolism, or food absorption) such as orlistat, and drugs interacting with green tea (e.g., digoxin, tacrolimus, warfarin).

Participants will be excluded from further analysis if they opt out, use antibiotics or other pre-, pro-, and synbiotic products, or meet the exclusion criteria anytime during the study, or do not comply with their assigned treatment regimen during the study.

### Who will take informed consent? {26a}

One of the research team members will be assigned to obtain informed consent from participants or their authorized representatives. To do this, a detailed ethics committee-approved consent form that explains the study’s purpose, procedures, risks, benefits, and the participant’s right to withdraw at any time will be provided to the participants in an understandable language and manner, allowing ample time for questions. Both the participant and the person obtaining consent will sign and date the document, and copies will be kept in trial records and given to the participant. We will only request surrogate consent if the participant has a disability.

### Additional consent provisions for collection and use of participant data and biological specimens {26b}

As part of this study, we will securely store de-identified participant data and blood samples in a coded form for potential use in future research that receives ethics committee approval and aligns with the original study’s ethical framework. These data and specimens will be stored at Mashhad University of Medical Sciences under strict confidentiality rules, accessible only to authorized researchers with ethics clearance. Participants can still opt out of future use of their data or specimens for other studies without affecting their current participation or rights, and they can request that stored materials be destroyed at any time. We may share anonymized data with other institutions for scientifically validated research upon institutional approval, but we will not disclose any personally identifiable information. Participants will not receive financial benefits from commercial uses of research findings (like patentable discoveries) unless required by law. We will publish all research outcomes in aggregate form to ensure confidentiality. If we want to recontact participants for additional studies, we will do so only under a separate ethics-approved protocol, with prior notice and consent options.

## Interventions

### Explanation for the choice of comparators {6b}

A placebo was chosen to isolate the effects of the supplement on the outcome variables. This approach enables a controlled evaluation of changes solely caused by the investigational supplement. The placebo will be given with identical appearance features to maintain participant blinding.

### Intervention description {11a}

A hypocaloric diet (500 kcal deficit) consisting of 50% carbohydrates, 20% protein, and 30% fat will be prescribed for participants in both groups. Total energy expenditure will be calculated based on the Mifflin equation. Participants will be encouraged to engage in 150 min of physical activity per week.

This study will utilize a supplement consisting of pasteurized *Akkermansia muciniphila*, green tea extract, and glucomannan konjac, offered in 2-g sachets. The placebo is formulated with microcrystalline cellulose and also comes in 2-g sachets. Participants will be instructed to consume one sachet 30 min before every meal (breakfast, lunch, and dinner) for 8 weeks. Parsi Lact Company (Shiraz, Iran) prepared and packaged the supplements and placebos in sachet form. Both the placebo and the supplement exhibit the highest level of similarity, and the containers will remain unlabeled and coded as A or B to ensure both researchers and participants are unaware of the type of intervention they will receive. Each participant will receive a monthly supply of the sachets they need for consumption over the course of a month. To promote sachet consumption, participants will receive weekly calls and briefing messages. Compliance will be tracked by counting the monthly returned used and unused sachets. Additionally, we will assess sachet consumption through food recalls. At the beginning of the study, all participants will complete a checklist to collect their general characteristics, medical history, and information regarding medications and supplements they are currently using.

### Criteria for discontinuing or modifying allocated interventions {11b}

For safety concerns, if a participant experiences severe or ongoing adverse effects (like unmanageable stomach symptoms or allergic reactions) due to the supplement, intervention will be stopped after discussing with a physician. The criteria for stopping or changing the assigned interventions include: (1) Safety concerns, such as severe adverse events (like anaphylaxis or severe gastrointestinal symptoms) or persistent intolerable reactions. (2) If a participant requests to stop at any time, their request will be *honored without consequence. (3) If a participant’s condition worsens, requiring the intervention to be terminated.*

### Strategies to improve adherence to interventions {11c}

Our strategies for increasing adherence to the intervention protocol include regular follow-ups through monthly in-person visits to review supplement use, dietary compliance, and symptom logs, along with weekly phone calls to monitor supplement use and adherence to the assigned intervention. Nutritionists will offer free counseling support, providing personalized feedback and motivation during these visits as an incentive. We will measure adherence through return rates, checklist completion, and self-reported logs. If participants miss more than 20% of doses, we will schedule a counseling session to address any barriers to adherence.

### Relevant concomitant care permitted or prohibited during the trial {11d}

Participants can take routine supplements like multivitamins within the dietary limits that do not affect the study outcomes. However, specific interventions are prohibited to maintain the trial’s integrity, including the use of prebiotic, synbiotic, and probiotic supplements and products. Participants are also prohibited from taking other weight-loss supplements, herbal products, or obesity medications, or participating in other conflicting clinical trials.

### Provisions for post-trial care {30}

According to informed consent, if any physical or mental problems occur to the participant during or after the research related to this research, the treatment of complications, their costs, and related compensation will be the responsibility of the supporting body.

### Outcomes {12}

#### Primary outcomes

##### Anthropometric measurements and assessment of body composition

In this study, the height and weight of participants will be recorded using standardized methods. Height will be measured with a wall-mounted stadiometer (Seca 206, Germany) to the nearest 0.1 cm, while weight will be taken with a digital floor scale (Seca 813, Germany) to the nearest 0.1 kg. Measurements will be obtained without shoes and in minimal clothing to ensure precision. The body mass index will be calculated by dividing the weight in kilograms by the square of the height in meters. Waist circumference will be measured using a flexible, non-stretchable tape measure at the midpoint between the bottom of the rib cage and the iliac crest, with participants standing upright, feet close together, and without any pressure applied. This measurement will be recorded to the nearest 1.0 cm. Hip circumference will be taken at the largest part of the pelvis using a non-stretchable tape. The waist-to-hip ratio will be computed using the appropriate formula. Body composition will be analyzed using the Tanita-MC780 body composition analyzer (Tanita Corporation, Tokyo, Japan). Instructions for participants before assessing body composition will be provided based on the manufacturer's suggestions (tanita.co.uk/mc-780ma-p). The instructions included abstaining from eating and drinking for three hours before performing the analysis, avoiding exercise and alcohol intake for at least 22 h, avoiding excess fluid intake 24 h before the analysis, and emptying the bladder before performing the analysis.

### Secondary outcomes

## Biochemical assessment

Biological specimens will be collected during the study. At both the start and conclusion of the study, 10 cc blood samples will be taken from all participants who have fasted for 12 h. Serum samples will be prepared by centrifuging the blood samples at 2500 rpm for 15 min and will be promptly stored at −20 °C until they are analyzed. The levels of fasting blood glucose, triglycerides, total cholesterol, high-density lipoprotein cholesterol, and low-density lipoprotein cholesterol will be assessed using the Biotecnica Instrument 1500 (BT 1500) analyzer and MAN, a commercial kit, following the guidelines provided by the manufacturer. Associated indices such as TYG, TC-HDL ratio, and FBS-HDL ratio will also be determined.

## Blood pressure measurement

Blood pressure will be measured at the start and end of the study. Participants’ systolic and diastolic pressures will be taken twice in the left arm with a digital device, allowing a 5-min interval between measurements. This happens while sitting following a 15-min break with a snug cuff. To ensure accuracy, participants must refrain from smoking, consuming coffee, or engaging in heavy exercise for 30 min before measurements. The average of the two readings will determine blood pressure.

## Assessment of appetite

The Visual Analogue Scale (VAS) in its online format will be utilized to evaluate subjective feelings of appetite. The VAS is a validated instrument that consists of a 100 mm horizontal line, marked with words at both ends that indicate the extremes of satiety, fullness, hunger, and prospective food consumption (PFC), along with the urge to eat sweet, salty, or fatty foods [21]. All patients will complete the VAS at the beginning, middle, and end of the study. The timing of the VAS assessments will occur before consumption of the sachet or meal, and at 30 min, 1 h, and 2 h after each meal.

### Further measurements

## Assessment of dietary intakes and physical activity levels

This study will evaluate dietary consumption using a three-day 24-h dietary recall, which will consist of two weekdays and one weekend, at the starting point and at the end of the fourth and eighth weeks, through in-person interviews. Furthermore, food recalls will be evaluated to determine the average daily macro- and micronutrient intakes of participants using Nutritionist IV software (First Databank Inc.), which has been customized for Iranian foods.

The levels of physical activity will be assessed at the beginning, in the fourth week, and in the last week by utilizing the short version of the International Physical Activity Questionnaire (IPAQ), which has been confirmed as valid for the Iranian population [22]. This questionnaire includes seven items and collects information on the time spent doing vigorous, moderate, and light activities in the past week. The questionnaire also gathers information on hours spent sitting. The questionnaire provides the quantitative metabolic equivalent (MET) per week.

## Assessment of gastrointestinal symptoms

The Gastrointestinal Symptom Rating Scale (GSRS) questionnaire will be utilized to assess potential gastrointestinal symptoms [23]. The GSRS consists of 15 items that measure the presence and intensity of gastrointestinal symptoms across five categories: abdominal pain, constipation, diarrhea, reflux, and indigestion. Each item on the questionnaire is rated on a 7-point Likert scale, where a score of one signifies no symptoms and a score of seven represents severe and intolerable symptoms. The Persian version of this questionnaire has already been validated. Participants from both groups will complete the GSRS at the start, mid-point, and conclusion of the study.

### Participant timeline {13}


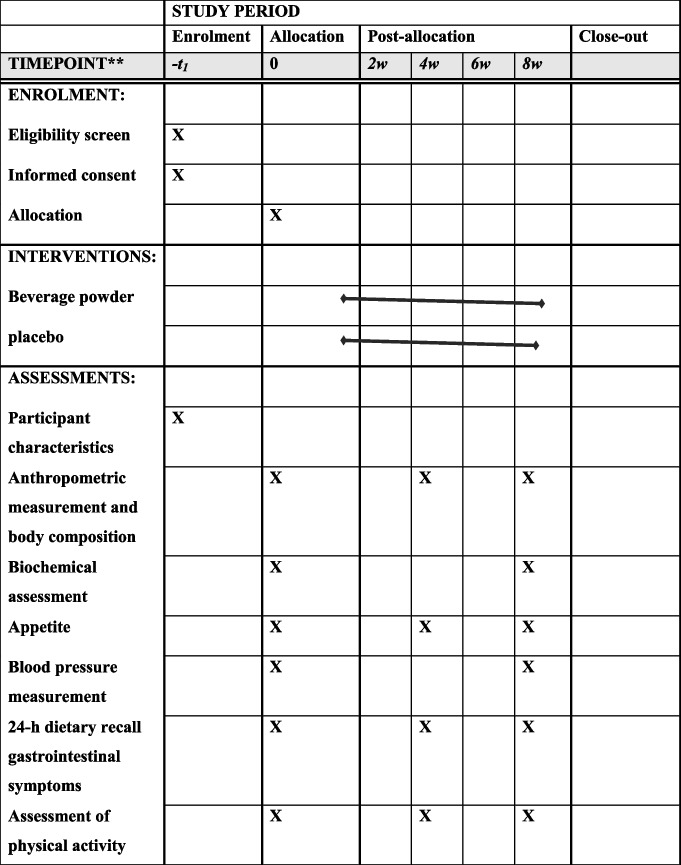


#### Sample size {14}

The sample size was calculated using the formula for the difference between two independent means, considering the mean and standard deviation of fat mass changes observed in the intervention (24.3 ± 1.8) and control (26.7 ± 2.8) groups as reported by D Roberts et al. [24]. With a type I error rate set at *α* = 0.05 and a statistical power of 1 − β = 0.90 aimed at detecting a standardized effect size of at least Δ = 0.80, the minimum required sample size for each group was determined to be 27 participants. However, after accounting for a potential dropout rate of 20%, this figure increased to 36 participants per group. Ultimately, a total of 72 overweight or obese adults will be recruited.

### Recruitment {15}

We will utilize the nutrition clinic to connect with potential participants and encourage their involvement. Before enrolling, we will screen for inclusion and exclusion criteria to minimize dropouts and ensure participants meet the study’s requirements. We will also offer flexible visit scheduling to accommodate participants’ schedules, increasing the likelihood of enrollment. Monthly, we will conduct a recruitment audit to monitor progress. If necessary, we will recruit participants from the university, comprehensive health care centers, and hospitals affiliated with Mashhad University of Medical Sciences to reach the target sample size.

## Assignment of interventions: allocation

### Sequence generation {16a}

Twelve blocks (each with a block size of 6) will be used to perform permuted block randomization for random allocation. Given the sample size of 72 subjects, the Random Allocation software will produce 12 blocks to form a set of sequentially numbered envelopes, each containing an equal number of assignments for either the placebo or intervention group. The study methodologist manages the study's randomization.

### Concealment mechanism {16b}

The pharmacist in charge of the study at Parsi Lact Company (Shiraz, Iran) manages the distribution of blinded placebos and supplements, which are labeled as A or B, identical in packaging, color, and taste. Allocation concealment will be carried out using sealed envelopes in the same order they were generated by the methodologist, who will be blinded to recruitment. The envelopes will be numbered and organized sequentially. For each patient enrolled in the study, an envelope following the generated sequence will be opened, and the patient will be assigned to either the control or the intervention group based on the envelope’s contents. Patient allocation and concealment will be performed by an individual not involved in the study.

### Implementation {16c}

Our study’s allocation sequence will be produced by the study methodologist using random allocation software to ensure participants are assigned fairly and randomly. The lead researcher will enroll participants after confirming their eligibility and obtaining informed consent. Once enrolled, someone not involved in the study will assign individuals to interventions, maintaining the integrity of the randomization process throughout the trial.

## Assignment of interventions: blinding

### Who will be blinded {17a}

After the interventions are assigned, participants, investigators, outcome assessors, and data analysts will remain blind to the allocations.

### Procedure for unblinding if needed {17b}

Unblinding will occur if a participant experiences a severe adverse event (SAE). To determine a participant’s assigned intervention, the designated team member will securely access the allocation code. This process will be documented to ensure transparency and accountability. After unblinding, the participant will receive appropriate medical care, and the incident will be reported to the relevant oversight bodies as required by regulations.

## Data collection and management

### Plans for assessment and collection of outcomes {18a}

Our study will put in place a thorough plan for gathering and assessing data, including baseline and other trial results. We will collect data using validated questionnaires and reliable lab methods that were presented in previous sections of the protocol. To maintain data quality, we will provide training for assessors and use duplicate measurements where possible. All data collection forms will be stored in a separate online repository, distinct from the primary trial documentation. We will regularly review the data to ensure accuracy and completeness.

### Plans to promote participant retention and complete follow-up {18b}

Measures will include frequent follow-up phone calls or social media messaging, tracking of alternate contact information (first of kin), and compliance monitoring for the supplement.

### Data management {19}

The use of double data entry and rechecking outliers will promote data quality. Data will be securely stored in a password-protected database to which access will be limited to the authorized personnel.

### Confidentiality {27}

To protect participant confidentiality, we will anonymize data using unique ID codes during collection. We will share anonymized data only with authorized persons. Anonymized electronic records will be stored on a Google Sheet, and paper records will be kept in secure facilities. After the trial, we will destroy identifiable data after 5 years and securely archive anonymized datasets for future research, pending ethics approval.

### Plans for collection, laboratory evaluation and storage of biological specimens for genetic or molecular analysis in this trial/future use {33}

At the start and after eight weeks, we take blood samples. We store these samples at −80 °C in a central biobank at Mashhad University of Medical Sciences, labeling them with anonymous codes for possible future use. All specimens will be stored for possible further studies in the university laboratory.

## Statistical methods

### Statistical methods for primary and secondary outcomes {20a}

All statistical analyses will be conducted using SPSS software version 26. The primary outcome is the change in anthropometric measures and body composition parameters (body weight, BMI, waist circumference, hip circumference, waist-to-hip ratio, fat mass, fat-free mass, and visceral fat) from baseline to the end of the intervention. The secondary outcome includes (1) changes in systolic and diastolic blood pressure, (2) biochemical indices including fasting blood glucose, triglycerides, total cholesterol, HDL cholesterol, LDL cholesterol, TYG index, TC-to-HDL ratio, and FBS-to-HDL ratio, and (3) appetite parameters derived from the VAS questionnaire (satiety, hunger, fullness, prospective food consumption, and desire for specific foods). The primary outcome will be evaluated at a two-sided significance level of *p* < 0.05. To account for multiple secondary outcomes and reduce the risk of type I error inflation, the Benjamini–Hochberg false discovery rate (FDR) adjustment will be applied.

Baseline characteristics between the two study groups will be compared using independent t-tests (or Mann–Whitney *U* tests for non-normally distributed data) for continuous variables and chi-square tests for categorical variables. For within-group changes over time, paired t-tests (or Wilcoxon signed-rank tests) will be used. Between-group comparisons of changes in primary and secondary outcomes will be performed using repeated measures ANOVA or ANCOVA, adjusting for baseline values and potential confounders (e.g., age, sex, baseline BMI), provided model assumptions are satisfied. If assumptions are violated, appropriate non-parametric alternatives will be applied. Post-hoc tests with Bonferroni correction will be conducted when global tests indicate statistical significance.

The normality of continuous variables will be assessed using the Shapiro–Wilk test and by visual inspection of histograms and Q-Q plots. Homogeneity of variances will be checked using Levene’s test. These steps are essential to determine whether parametric or non-parametric statistical methods should be applied.

### Interim analyses {21b}

There are no plans for interim analyses because the intervention presents a low risk. The trial will proceed as scheduled unless serious adverse events warrant early termination, in which case the principal investigator will decide in consultation with the ethics committee.

### Methods for additional analyses (e.g. subgroup analyses) {20b}

There are currently no plans to conduct subgroup analyses in this trial.

### Methods in analysis to handle protocol non-adherence and any statistical methods to handle missing data {20c}

All analyses will follow the intention-to-treat (ITT) principle. Missing data will be addressed using multiple imputation under the assumption of missing at random (MAR). Anthropometric measures, body composition variables, blood pressure, biochemical indices, and questionnaire scores will be included in the imputation model using a fully conditional specification (FCS) approach. In addition, sensitivity analyses will be performed with complete cases to confirm the robustness of the results.

### Plans to give access to the full protocol, participant level-data and statistical code {31c}

Our complete trial protocol will be published in a peer-reviewed journal. We will make anonymized participant-level data and statistical code available upon a reasonable request from the corresponding author, following our data sharing policies and after getting the necessary ethical approvals.

## Oversight and monitoring

### Composition of the coordinating centre and trial steering committee {5d}

In accordance with university regulations, one member of the reviewing panel (made up of independent experts in nutrition, biostatistics, and ethics who are not affiliated with the sponsor) from the university Ethical Committee is elected to assess the adherence to the research proposal and monitor the correctness of data analysis. According to university regulations, once 5% of the sample size data has been gathered, the inspector will evaluate the consistency between the proposal and the data collection and intervention processes. If any complications arise that disrupt the study or lead to serious side effects, the study will be stopped, and these issues will be reported to the Ethical Committee. Participants will be urged to inform the researcher of any adverse events experienced in connection with the intervention supplement. The researcher will then evaluate the reported adverse event alongside the research team, and should a connection between the supplement and the adverse event be established, it will be documented. If the adverse event becomes too intolerable for a participant, they will have the option to withdraw from the study.

### Composition of the data monitoring committee, its role and reporting structure {21a}

If additional assessment is necessary, a Data Monitoring Committee (DMC)—made up of the Ethical Committee members who reviewed the proposal, along with supplementary experts for specific evaluations—will be convened. The study inspector will provide reports to the Ethical Committee. Throughout the study, the inspector is permitted to perform random checks on the procedures without notifying the research team. The inspector will maintain no financial or conflicting interests with either the sponsor or the research groups involved. The committee has no financial or professional connection to the sponsor and will conduct unannounced audits to ensure the trial adheres to ethical and methodological standards.

### Adverse event reporting and harms {22}

Participants will stay in contact with the researchers once a week, and any issues or adverse effects that arise during the intervention will be discussed. If any adverse side effects or problems are identified, participants will be removed from the study after consulting with the research team and specialists in the relevant field. We follow institutional guidelines for reporting and managing adverse effects.

### Frequency and plans for auditing trial conduct {23}

The university’s clinical trial monitoring protocol mandates strict adherence to approved ethical and methodological standards, overseen by a three-member supervisory team specified in the research contract. These independent auditors from the ethics committee, with no association with the research team or sponsor, will conduct audits and perform unannounced random checks at least twice during the trial. Additionally, the sponsor will have no role in designing, executing, or interpreting audits. Investigators must notify the Research Administration when reaching 5% of the target sample size to coordinate field audits, which are scheduled on predetermined days, with the research team proposing two alternative times. During audits, the supervisory team evaluates compliance with protocols, including sampling methods, randomization, blinding, data integrity, informed consent documentation, and confidentiality measures. Investigators are responsible for ensuring staff availability to provide requested records and answer questions. Deviations from the approved protocol trigger a structured escalation process: initial non-compliance results in a corrective action period, followed by a re-audit. Persistent deviations may lead to referral to the Clinical Trials Committee and possible study termination by the Research Council. This process, overseen by the Vice Chancellor for Research and Technology, emphasizes transparency, accountability, and institutional oversight, with all audit results documented to maintain traceability and uphold research integrity.

### Plans for communicating important protocol amendments to relevant parties (e.g., trial participants, ethical committees) {25}

Any significant protocol changes will be submitted for approval to the Ethical Committee of Mashhad University of Medical Sciences before they are implemented. Approved amendments will be communicated to all investigators and documented in IRCT.

### Dissemination plans {31a}

The study results will be published in relevant publications. Access to the data will begin six months after the results are published. The non-identifiable personal information of the participants will then be available to other researchers at academic institutions upon request to the corresponding authors. It may only be used for research purposes. The trial results will also be updated on the IRCT.

## Discussion

Obesity and its related disorders present a significant public health challenge globally. In addition to traditional weight loss medications, next-generation probiotics (NGPs) appear to hold promise as a preventive and treatment measure against obesity [25]. Emerging research is investigating the benefits of next-generation probiotic supplements, with a focus on the effects of *Akkermansia muciniphila* on metabolic conditions such as obesity and diabetes.

Although *Akkermansia muciniphila* shows potential for treating obesity and type 2 diabetes (T2D), human research is scarce [26]. Further human studies could better clarify the preventive or therapeutic benefits of this promising next-generation probiotic.

Although studies have focused on the effectiveness of live or pasteurized *Akkermansia muciniphila,* the effect of this bacterium in combination with prebiotics has not yet been investigated. Filling this gap could result in innovative treatments for metabolic conditions, including obesity. The purpose of this study is to investigate the effect of a novel beverage containing pasteurized *Akkermansia muciniphila*, glucomannan, and green tea extract on body composition, anthropometric indices, metabolic markers, blood pressure, and appetite in adults with overweight or obesity. If the intervention shows favorable outcomes, it will represent a valuable step toward developing recombinant supplements with specific therapeutic targets.

## Trial status

The current protocol version is 4.0, dated September 5, 2025. Recruitment for the study began on June 22, 2025, and it is estimated that recruitment will be completed by October 23, 2025.

## Supplementary Information

Additional file 1

## Data Availability

Data from the main study will be available after obtaining institutional approval upon written request.

## Abbreviations

*A. muciniphila*: *Akkermansia muciniphila*
BP: Blood pressure
BMI: Body mass index
CKD: Chronic kidney disease
CVD: Cardiovascular diseases
DMC: Data Monitoring Committee
EGCG: Epigallocatechin-3-gallate
FBS: Fasting blood sugar
GSRS: Gastrointestinal Symptom Rating Scale
HDL: High-density lipoprotein
IRCT: Iranian Registry of Clinical Trials
KGM: Konjac Glucomannan
MET: Metabolic Equivalent of Task
NAFLD: Nonalcoholic fatty liver disease
NGPs: Next-generation probiotics
SPSS: Statistical Package for the Social Sciences
TC: Total cholesterol
LDL: Low-density lipoprotein
LDL-C: Low-density lipoprotein cholesterol
T2D: Type 2 diabetes
T2DM: Type 2 diabetes mellitus
VAS: Visual Analogue Scale
WHO: World Health Organization
MetS: Metabolic syndrome
